# The highest altitudinal record of the Mountain coati *Nasuella olivacea* (Gray, 1865) for Ecuador

**DOI:** 10.3897/BDJ.6.e29160

**Published:** 2018-12-14

**Authors:** Pablo Medrano-Vizcaíno

**Affiliations:** 1 Proyecto Paisajes Vida Silvestre - Ministerio del Ambiente del Ecuador – Programa de las Naciones Unidas para el Desarrollo, Quito, Ecuador Proyecto Paisajes Vida Silvestre - Ministerio del Ambiente del Ecuador – Programa de las Naciones Unidas para el Desarrollo Quito Ecuador; 2 Centro de Biología, Universidad Central del Ecuador, Quito, Ecuador Centro de Biología, Universidad Central del Ecuador Quito Ecuador

**Keywords:** cuchucho, Llanganates National Park, paramo, range extension, Procyonidae

## Abstract

The Mountain coati *Nasuella olivacea* is a species of carnivorous mammal, several aspects of its ecology and natural history remain unknown. In Ecuador, its presence is reported in high Andean forest and paramo between 1,300 and 3,700 m a.s.l., being rare over 3,200 m. In this work, I present the extension of its altitudinal limit for Ecuador to 3,862 m a.s.l.; in addition, I report an event of a possible interaction between *Tremarctos ornatus* and *N. olivacea*.

## Introduction

The Mountain coati *Nasuella olivacea*, also known as cuchucho or andasolo, is a carnivore species that belongs to the Procyonidae family; it is very similar to the lowland coati (*Nasua nasua*), but a little smaller (Tirira 2017). It weighs between 1 and 1.5 kg, body and head length can reach 50 cm while tail can get to 30 cm. This mammal shows an olive-brown fur with a whitish base, dark edge of the eyes, black legs and dark tail rings (Albuja et al. 2012).

*N. olivacea* is a diurnal, terrestrial, arboreal and gregarious species, only adult males being solitary (Tirira 2017, Vallejo 2017). Its diet is omnivorous, based on vegetables, fruits, vertebrates and invertebrates, showing a preference for the consumption of Coleoptera, Orthoptera, Myriapoda and Hymenoptera insects (Rodríguez-Bolaños et al. 2000).

It is a rare species and information about ecology and natural history is unknown, but it could be similar to *Nasua nasua* (Tirira 2017).

According to the red book of mammals of Ecuador, it is a vulnerable species. Main causes for this category are deforestation, expansion of the agricultural frontier, roadkills and hunting. These are focal threats for populations survival (Tirira 2011, González-Maya et al. 2016).

For Ecuador, its presence range is about 20,000 km^2^ along fragmented areas. This factor obviously affects genetic flow amongst populations and reduces its viability (Tirira 2011).

This species is distributed along the Andes of Colombia and Ecuador, specifically in highlands and paramo areas (Helgen et al. 2009, Balaguera-Reina et al. 2009). In Ecuador, it is found in Imbabura, Carchi, Pichincha, Cotopaxi, Bolívar, Tungurahua, Chimborazo, Cañar, Azuay, Loja and Napo provinces (Vallejo 2017). In this manuscript, I present new distribution data for Ecuador and a possible feeding interaction between the spectacled bear (*Tremarctos ornatus*) and *N. olivacea*. These data were obtained from fieldwork carried out by the Project Paisajes Vida Silvestre (Ministry of Environment of Ecuador).

## Materials and Methods

**Figure 1.**
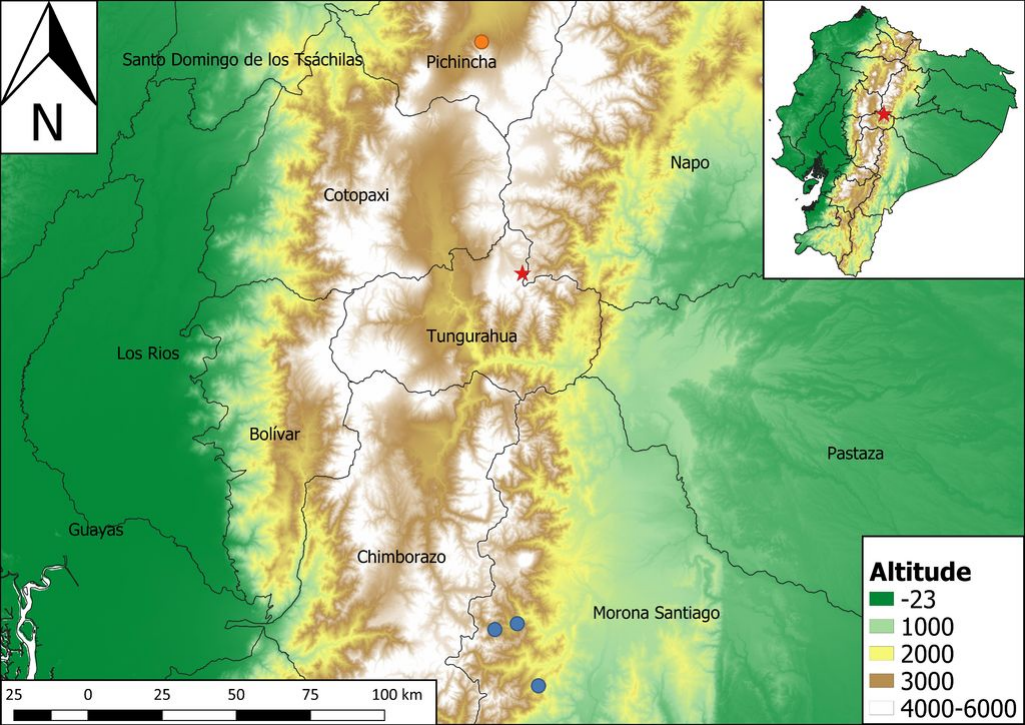
Records for *Nasuella olivacea* in Ecuador. Red star: this record (3,862 m a.s.l.); Orange circle: Previous highest altitudinal report with a supporting document for Ecuador (3,357 m a.s.l.) (Ramírez 2011); Blue circles: Other records reported for Ecuador (2,300; 2,500 and 2,800 m a.s.l.) (Brito and Ojala-Barbour 2016)

For twelve days, between 4 October and 15 October 2017, together with people from the Ministry of Environment, we conducted a sampling expedition of medium and large mammals to the buffer zone of Llanganates National Park, located in Tungurahua and Pastaza provinces (Fig. 1). We worked by installing camera traps and looking for direct and indirect signs (hair, faeces, bones and footprints) of wildlife presence.

*N. olivacea* bones were found in a paramo area surrounded by scrublands. I registered geographical coordinates and altitude with a Global Positioning System (GPS) Garmin GPSMAP 64S.

This research was carried out under authorisation permission from the Ministry of Environment N°019-17LC-FAU-DNB/MA.

### Identification

Skull identification was confirmed considering morphological characteristics described by Helgen et al. (2009) (Fig. 3a, b, c). The only similar species in Ecuador is *Nasua nasua*, but it is not present in the same distribution area. *N. nasua* inhabits humid, dry, tropical and subtropical forests between 200 and 1,800 m a.s.l. opposite to *N. olivacea* that inhabits highlands and paramo areas (Tirira 2017, Balaguera-Reina et al. 2009).

## Results

On 12 October 2017 at 11:47 am, in the Province of Tungurahua, Marcos Espinel Parish, Sunfopamba locality (-78.35196W, -1.13237S) (Fig. 1) while looking for direct and indirect signs of wildlife, I found Andean coati bones, in a paramo area at 3,862 m a.s.l., in a place surrounded by scrublands.

**Figure 2.**
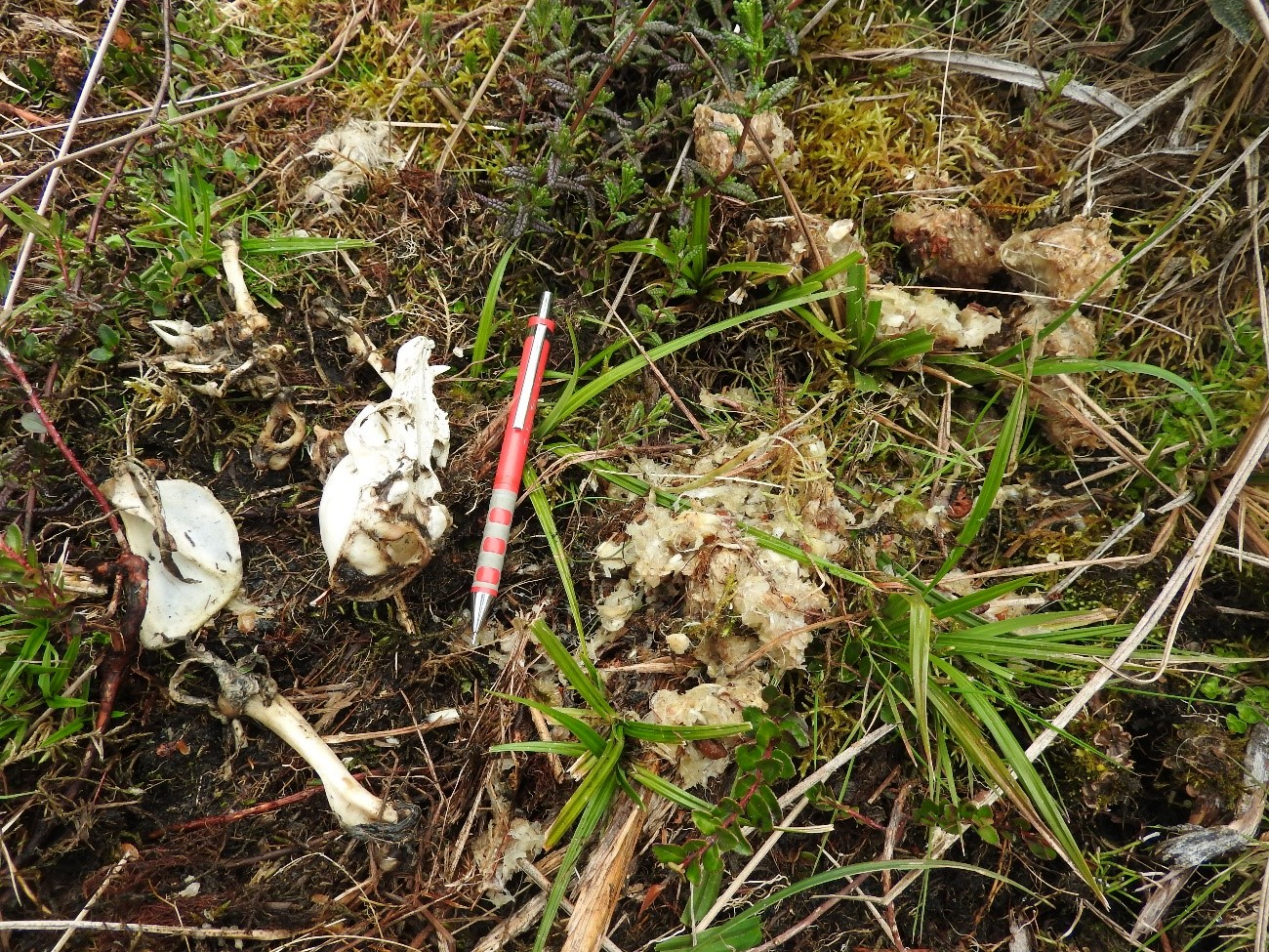
*Nasuella olivacea* bones near bear faeces.

As complementary information, it is important to add that coati bones were found near spectacled bear (*Tremarctos ornatus*) faeces (Fig. 2)

## Discussion

Helgen et al. (2009) mention that *N. olivacea* dwells between 1,300 and 4,250 m a.s.l. in Colombia and Ecuador, nevertheless they do not report the country where this altitudinal limit belongs to. The highest altitudinal records with scientific literature support for Colombia belong to: Castaño et al. (2003), they registered this species between 1,700 and 4,100 m a.s.l., meanwhile, Andrade Ponce et al. (2016) reported at 3,500 m a.s.l. On the other hand, records above 3,200 m a.s.l. are rare for Ecuador (Tirira 2017). Although this last author mentions that this species is found in Ecuador between 1,700 m a.s.l. and 3,700 m a.s.l., there is no information about data collection, nor scientific literature supporting this altitudinal limit. The prior highest altitudinal report with a supporting document for Ecuador corresponds to Ramírez (2011), who found this species at 3,337 m a.s.l. in Pasochoa volcano. Other records were found at 2,300, 2,500 and 2,800 m a.s.l. in Sangay National Park (Brito and Ojala-Barbour 2016) (Fig. 1). Therefore, the altitudinal limit presented in this work is the highest for Ecuador (3,862 m a.s.l.).

Furthermore, because the Andean bear (*T. ornatus*) is an opportunistic feeder and may be a scavenger and a predator (Cavelier et al. 2010), closeness between bear faeces and coati bones open some possibilities such as: 1) *T. ornatus* was feeding on coati remains (scavenger habits), 2) *T. ornatus* hunted Andean coati (predator habits) as it has already been reported that the spectacled bear is capable of hunting agile animals like rabbits *Sylvilagus brasiliensis* (Castellanos 2011) or 3) there is no relationship between bear faeces and coati bones.

It is important to highlight that coati bones were not found in any sample of bear faeces while conducting fieldwork, therefore, a reasonable answer would be that coati bones are not related to faeces. However, because there is not enough evidence to confirm or discard one of the possibilities mentioned in the last paragraph, it is interesting to show possible interactions between these two species.

Many aspects of ecology and natural history of *N. olivacea* are unknown (Tirira 2017), therefore, it is extremely necessary to publish new information to enlarge the knowledge of this species. This article contributes new information about distribution and possible feeding interactions between the Mountain coati and the Andean bear.

## Figures and Tables

**Figure 3a.**
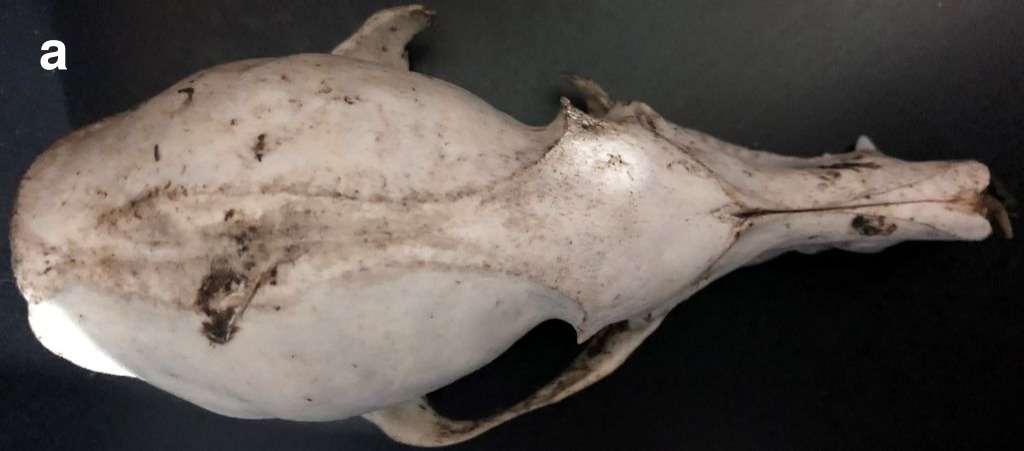
Dorsal view.

**Figure 3b.**
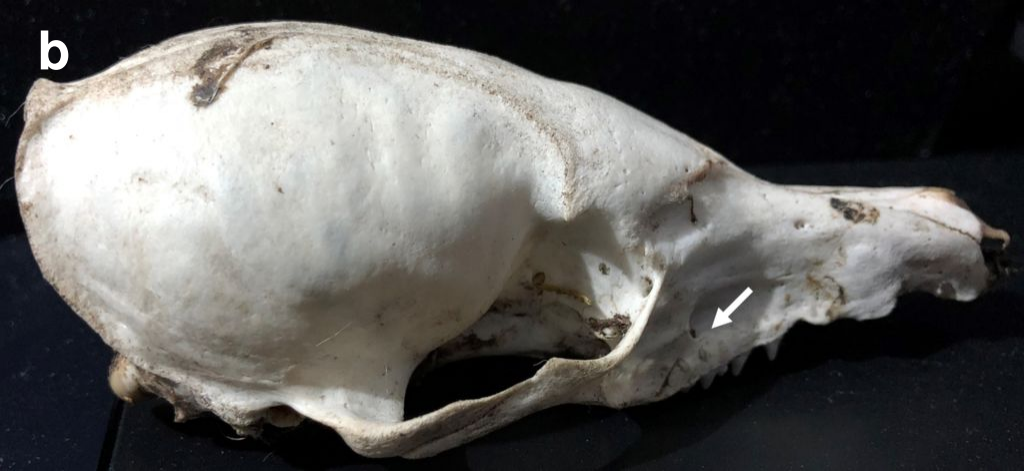
Lateral view: White arrow shows the position of the anterior alveolar foramen.

**Figure 3c.**
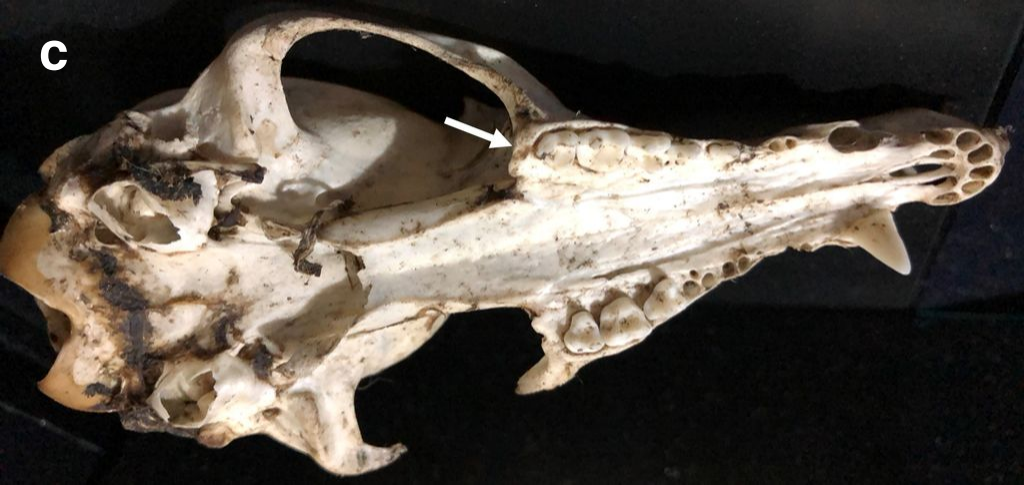
Ventral view: White arrow shows the palate behind the last molar.
